# MUC22, HLA-A, and HLA-DOB variants and COVID-19 in resilient super-agers from Brazil

**DOI:** 10.3389/fimmu.2022.975918

**Published:** 2022-10-25

**Authors:** Erick C. Castelli, Mateus V. de Castro, Michel S. Naslavsky, Marilia O. Scliar, Nayane S. B. Silva, Raphaela N. Pereira, Viviane A. O. Ciriaco, Camila F. B. Castro, Celso T. Mendes-Junior, Etiele de S. Silveira, Iuri M. de Oliveira, Eduardo C. Antonio, Gustavo F. Vieira, Diogo Meyer, Kelly Nunes, Larissa R. B. Matos, Monize V. R. Silva, Jaqueline Y. T. Wang, Joyce Esposito, Vivian R. Cória, Jhosiene Y. Magawa, Keity S. Santos, Edecio Cunha-Neto, Jorge Kalil, Raul H. Bortolin, Mário Hiroyuki Hirata, Luiz P. Dell’Aquila, Alvaro Razuk-Filho, Pedro B. Batista-Júnior, Amaro N. Duarte-Neto, Marisa Dolhnikoff, Paulo H. N. Saldiva, Maria Rita Passos-Bueno, Mayana Zatz

**Affiliations:** ^1^ Department of Pathology, School of Medicine, São Paulo State University (UNESP), Botucatu, Brazil; ^2^ Molecular Genetics and Bioinformatics Laboratory, Experimental Research Unit (Unipex), School of Medicine, São Paulo State University (UNESP), Botucatu, Brazil; ^3^ Human Genome and Stem Cell Research Center, University of São Paulo, São Paulo, Brazil; ^4^ Department of Genetics and Evolutionary Biology, Biosciences Institute, University of São Paulo, São Paulo, Brazil; ^5^ Centro Universitário Sudoeste Paulista, Avaré, Brazil; ^6^ Departamento de Química, Faculdade de Filosofa, Ciências e Letras de Ribeirão Preto, Universidade de São Paulo, Ribeirão Preto, Brazil; ^7^ Programa de Pós-Graduação em Genética e Biologia Molecular, Universidade Federal do Rio Grande do Sul (UFRGS), Porto Alegre, Brazil; ^8^ Laboratório de Saúde Humana In Silico, Programa de Pós-Graduação em Saúde e Desenvolvimento Humano, Universidade La Salle, Canoas, Brazil; ^9^ Departamento de Clínica Médica, Disciplina de Alergia e Imunologia Clínica, Faculdade de Medicina da Universidade de São Paulo, São Paulo, Brazil; ^10^ Laboratório de Imunologia, Instituto do Coração (InCor), LIM19, Hospital das Clínicas da Faculdade de Medicina da Universidade de São Paulo, (HCFMUSP), São Paulo, Brazil; ^11^ Instituto de Investigação em Imunologia, Instituto Nacional de Ciências e Tecnologia-iii (INCT), São Paulo, Brazil; ^12^ Department of Clinical and Toxicological Analyses, School of Pharmaceutical Sciences, University of São Paulo, São Paulo, Brazil; ^13^ Prevent Senior Institute, São Paulo, Brazil; ^14^ Department of Pathology, School of Medicine, University of Sao Paulo, Sao Paulo, Brazil

**Keywords:** human leukocyte antigens, immune response, major histocompatibility complex (MCH), HLA, SARS-CoV-2, MUC22, COVID-19, resistant genetic variants

## Abstract

**Background:**

Although aging correlates with a worse prognosis for Covid-19, super elderly still unvaccinated individuals presenting mild or no symptoms have been reported worldwide. Most of the reported genetic variants responsible for increased disease susceptibility are associated with immune response, involving type I IFN immunity and modulation; *HLA* cluster genes; inflammasome activation; genes of interleukins; and chemokines receptors. On the other hand, little is known about the resistance mechanisms against SARS-CoV-2 infection. Here, we addressed polymorphisms in the MHC region associated with Covid-19 outcome in super elderly resilient patients as compared to younger patients with a severe outcome.

**Methods:**

SARS-CoV-2 infection was confirmed by RT-PCR test. Aiming to identify candidate genes associated with host resistance, we investigated 87 individuals older than 90 years who recovered from Covid-19 with mild symptoms or who remained asymptomatic following positive test for SARS-CoV-2 as compared to 55 individuals younger than 60 years who had a severe disease or died due to Covid-19, as well as to the general elderly population from the same city. Whole-exome sequencing and an in-depth analysis of the MHC region was performed. All samples were collected in early 2020 and before the local vaccination programs started.

**Results:**

We found that the resilient super elderly group displayed a higher frequency of some missense variants in the *MUC22* gene (a member of the mucins’ family) as one of the strongest signals in the MHC region as compared to the severe Covid-19 group and the general elderly control population. For example, the missense variant rs62399430 at *MUC22* is two times more frequent among the resilient super elderly (p = 0.00002, OR = 2.24).

**Conclusion:**

Since the pro-inflammatory basal state in the elderly may enhance the susceptibility to severe Covid-19, we hypothesized that *MUC22* might play an important protective role against severe Covid-19, by reducing overactive immune responses in the senior population.

## Introduction

Although a diverse clinical spectrum has been described among patients with Covid-19, robust data show that increasing age correlates with more severe disease and a higher frequency of deaths worldwide (1). While the elderly have a higher prevalence of comorbidities such as cardiovascular diseases, diabetes, and cancer - which are also independently associated with a higher risk of severe Covid-19 (2), increasing age is still the most significant risk factor for Covid-19 mortality (3). To yield clues about this infection susceptibility, the comparison of extremely older people presenting mild symptoms and young adults with a very severe outcome may contribute with relevant observations.

Covid-19 severity among the elderly may be related to immunosenescence, changes in cytokine patterns, activation of inflammatory pathways, and impaired innate and adaptive immune responses (4–6). Also, comorbidities in older individuals are strongly associated with an increased risk of Covid-19 complications (7, 8). Alternatively, older individuals are more likely to have been exposed to other corona and influenza viruses during their lifespan, even by vaccination, increasing their odds of defeating SARS-CoV-2 (9). For instance, centenarians exposed to the 1918 H1N1 influenza virus might present some protection against SARS-CoV-2 infection (10).

The MHC (Major Histocompatibility Complex) region contains more than 200 genes, many of them related to immunity. Therefore, it is a natural candidate for influencing infectious disease susceptibility and severity. MHC genes influence different levels of the immune response against viruses, such as genes encoding cytokines and molecules of the complement system, which may influence Covid-19 severity and cytokine storm (11, 12), genes encoding membrane-associated mucins (*MUC22*) (13), and genes encoding molecules that mediate NK cell responses (*HLA-G*, *HLA-E*, *MICA*, and *MICB*) (14–17). Some of the MHC genes are particularly important to the antigen presentation pathway. *HLA-A*, *HLA-B*, and *HLA-C* encode the heavy chain of the MHC-I molecule, responsible for binding the intracellular antigens and presenting them on the cell surface to the T cell receptor (TCR) of CD8 T lymphocytes. Likewise, *HLA-DRA*, *HLA-DRB1*, *HLA-DQA1*, *HLA-DQB1*, *HLA-DPA1*, *HLA-DPB1*, and others, encode the MHC-II molecule, responsible for binding the exogenous antigens, usually internalized by antigen-presenting cells such as macrophages, and presenting them on the cell surface to CD4 T lymphocytes. These genes are highly polymorphic, with hundreds to thousands of alleles for each locus (18). Such diversity influences antigen presentation since different MHC molecules may present a different subset of antigens (19).

Because of the unusually high polymorphism and extensive paralogy of the MHC region, particularly at the HLA classical class I and II genes, the MHC region requires specialized tools to align short reads correctly and, thus, call genotypes and haplotypes properly (20, 21). Additionally, allele frequencies vary across populations, as clearly documented in previous studies focusing on HLA genes and Covid-19 (22–26). Associations between HLA genotype and disease severity extend to other unrelated viruses, such as HIV and dengue (27, 28). Some MHC variants have already been reported to be associated with Covid-19 severity. *HLA-G* variant rs9380142 was associated with Covid-19 critical illness (29) and *HLA-E* allele E*01:01 with Covid-19 severity, particularly in patients requiring intensive care (30). The *CCHCR1* locus was associated with critical illness in Covid-19 (29).

We hypothesized that differences in MHC can predispose to a severe or mild clinical course in Covid-19 despite age. Therefore, aiming to verify the existence of MHC differences between extreme opposed outcomes for Covid-19, we have analyzed exomes of Brazilian convalescents by SARS-COV-2 grouped according to Covid-19 clinical status and age: a group of super elderly patients (>= 94 yo) recovered from Covid-19 with mild to moderate symptoms (without ventilation support) as compared to younger adults (mean age <= 52) with severe disease (with ventilation support). We also compared patients with a previously whole-genome sequenced (WGS) census-based sample of elderly individuals from the same city (São Paulo, Brazil), sampled before the current pandemic (31). Brazilians are a highly admixed population composed of tri-hybrid proportions of European (average 73%), African (18%), and Native American (9%) ancestries (31). This was taken into account and therefore global genomic ancestry was controlled when performing the association study.

## Materials and methods

### Definition of the groups

**Figure 1:**
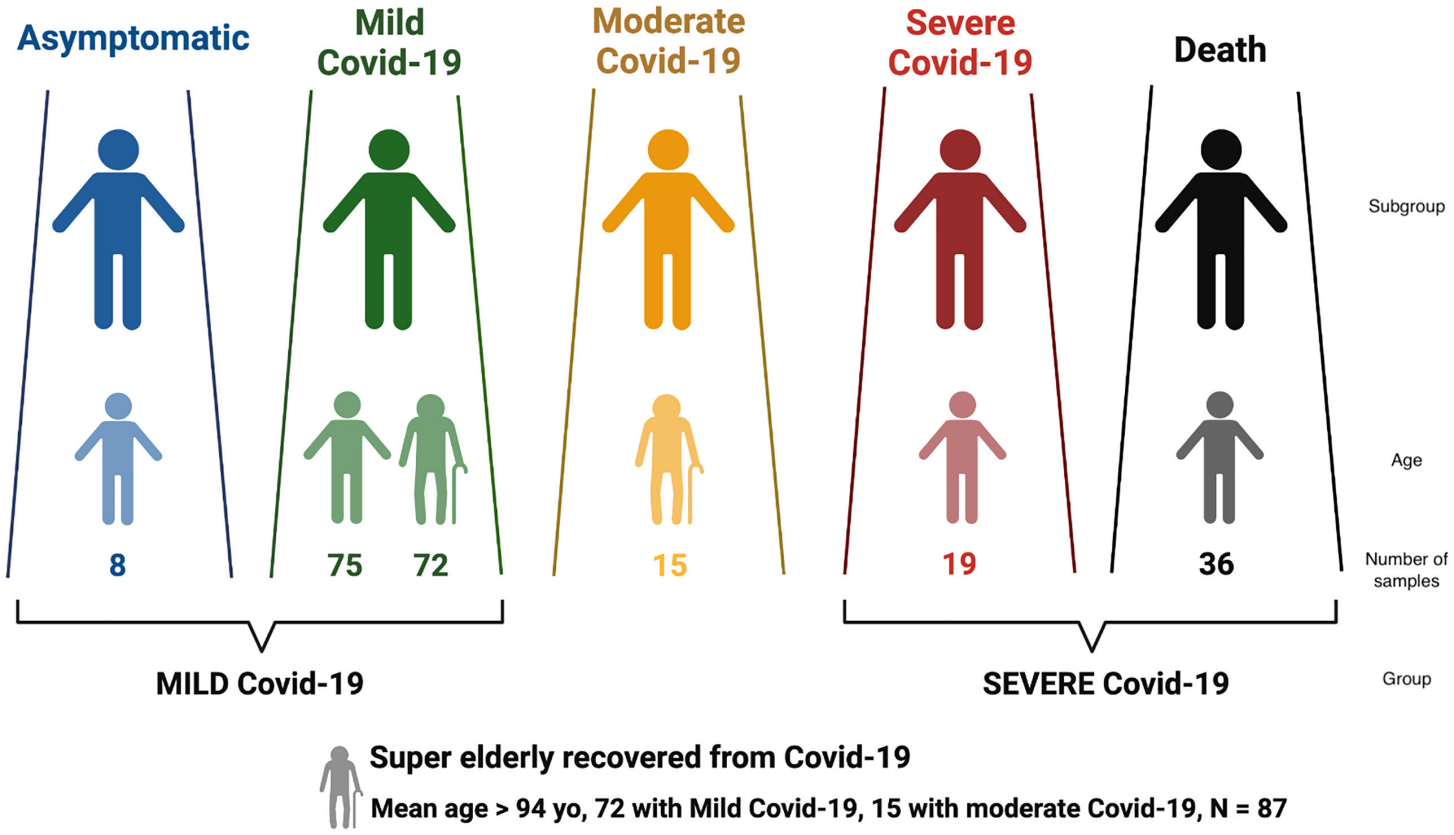
Distribution of Brazilian individuals with SARS-CoV-2 infection according to Covid-19 severity, and the definition of the larger groups MILD and SEVERE Covid-19.

This survey included 225 patients with Covid-19, as illustrated in **Figure 1**. Diagnostic tests (RT-PCR) confirmed the positive SARS-CoV-2 infection in all individuals. The samples were collected between June and October 2020, before new SARS-CoV-2 variants were reported in Brazil (especially Gamma) and before the onset of the Brazilian vaccination program against Covid-19.

Covid-19 severity was classified according to the clinical spectrum of the World Health Organization’s updated guideline for Covid-19 treatment (https://www.covid19treatmentguidelines.nih.gov/overview/clinical-spectrum/). Patients that were asymptomatic or presented mild symptoms were grouped into MILD Covid-19; deceased and/or hospitalized in ICU requiring ventilation support were grouped into SEVERE Covid-19 (**Figure 1**). The SEVERE group is significantly younger than the MILD group (**Supplementary Table S1**, *p*< 10^-5^).

We also considered a special group named super elderly (>= 94 yo) who recovered from Covid-19, with 72 super elderly with mild Covid-19 and 15 with moderate symptoms. We retrieved clinical data regarding the progression of Covi-19 and diagnostic test results.

These Covid-19 groups were compared with MHC data from a previously whole-genome sequenced (WGS) sample of Brazilian elderly individuals (>= 65 yo), known as the SABE cohort (31), collected before the SARS-CoV-2 outbreak and representative of the general elderly population in the same city. Age, sex, and mean genetic ancestry distributions for each group are displayed in **Supplementary Table S1** and were used as co-variables in regression models.

### Exome and whole-genome sequencing

We obtained full exomes from DNA extracted from patients’ samples with SARS-CoV-2 infection. We used the Nextera Rapid Capture Custom Enrichment Kit or the Nextera Flex Kit (Illumina, San Diego, CA, USA) for library preparation and the IDT xgen-V1 kit for capture following manufacturer protocols. Whole-exome sequencing was performed on the NovaSeq 6000 equipment (Illumina, USA) with a 150-base paired-end dual index read format. Reads were aligned to the human reference GRCh38 using Burrow–Wheeler Aligner (BWA), algorithm MEM (https://github.com/lh3/bwa/tree/master/bwakit). We also called genotypes using GATK HaplotypeCaller (version 4.0.9). We used the genotypes obtained in this step to infer the genetic ancestry. The pipeline used for alignment, variant calling, variant refinement, and genetic ancestry assessment is detailed elsewhere (22).

For the general elderly population (SABE), whole-genome sequencing was performed previously (31). Although SARS-Cov-2 infection is unknown for this cohort, this data provides a baseline for the frequency of each polymorphism in the general elderly population from São Paulo.

### MHC genotyping and haplotyping

The MHC region is prone to genotyping errors because of alignment bias in paralogous and highly polymorphic genes (20, 21, 32). The HLA classical class I and II genes are the most impacted ones by alignment bias, and conventional NGS analysis workflows are not suitable for genotyping them. We used a customized workflow to circumvent this issue and get reliable genotypes and haplotypes in the MHC region. We used HLA-mapper (version 4) (20) to optimize read alignment along the MHC region (**Supplementary Figure S1**). The input for HLA-mapper is the BAM file obtained in the previous step (for exomes or whole-genomes). After applying HLA-mapper to correct the alignments, we called genotypes using GATK 4 HaplotypeCaller. Then, we selected only the variants that overlapped the region captured by the Exome, with no more than 5% of missing alleles in the Exomes. After, we refined the variants using the standard Variant Quality Score Recalibration (VQSR) supplemented with known variants from HLA genes. To obtain phased variants for each gene, we first phased closely located variants using WhatsHap (33). Then, we combined phase sets using Shapeit 4 (34). The final product is a phased VCF with SNPs throughout the MHC.

### HLA allele calling

For *HLA-A*, *HLA-B*, *HLA-C*, *HLA-E*, *HLA-F*, *HLA-G*, *MICA*, *MICB*, *HLA-DOA*, *HLA-DOB*, *HLA-DMA*, *HLA-DMB*, *HLA-DRA*, *HLA-DPA1*, *HLA-DPB1*, *TAP1*, and *TAP2*, we obtained the complete exonic sequences for each individual by converting the phased VCF obtained in the previous step into complete CDS sequences using vcfx transcript (www.castelli-lab.net/apps/vcfx). We also translated these sequences into protein sequences (the allotypes) using Emboss transeq. We called HLA alleles (3-field and 2-field resolution) directly from these CDS and predicted protein sequences, comparing them with the ones reported in the IPD-IMGT/HLA database (35). Because of exome probe-capturing bias in some MHC regions, we imputed *HLA-DRB1*, *HLA-DQA1*, and *HLA-DQB1* 2-field alleles instead of calling alleles directly from the VCF data, as discussed in the next section.

### Quality control for MHC variants and HLA imputation for *HLA-DRB1*, *HLA-DQA1*, and HLA*-DQB1*

For exomes, we noticed a probe-capturing bias in some MHC regions, which is not an unexpected issue, especially for HLA genes (32, 36). This bias is quite strong for *HLA-DRB1*, *HLA-DQA1*, and *HLA-DQB1*, as illustrated in **Supplementary Figure S2**. Although the HLA-mapper optimization corrects most of the alignment errors in HLA genes, the absence of sequences from one chromosome (the capture bias) led to genotyping bias and allele call errors. This is particularly problematic when comparing exomes and whole genomes because this error occurs only in the former.

To circumvent this issue, we only selected from the exomes the variants with an average proportion of reads among alleles in heterozygous sites (i.e., the allele balance) over 0.3, represented as a red line in **Supplementary Figure S2**. This procedure might have eliminated some important variants, but it allowed us to compare exomes and whole genomes by avoiding variants prone to genotyping errors. In addition, we selected only variants with a frequency of at least 1%, either among patients or among the general elderly population. Accordingly, 2,346 SNPs were selected across the MHC and were considered for all subsequent analyses.

The capture bias discussed above prevented the direct call of HLA alleles for *HLA-DRB1*, *HLA-DQA1*, and *HLA-DQB1*. We applied an imputation method with HIBAG 1.5 (37) to call 2-field resolution alleles (**Supplementary Figure S1**). First, we built a reference panel based on whole-genome data from Brazilians, the 1000Genomes, and HGDP datasets, using the same pipeline as presented in **Supplementary Figure S1**. The selected variants for the imputation model are bi-allelic variants present in both the reference panel and exomes and show an average proportion of reads among alleles in heterozygous sites over 0.30 in exomes (**Supplementary Figure S2**). After imputation, the incompatibility between imputed HLA alleles and direct calls was as follows: HLA-A (4.1%), HLA-B (2.8%), HLA-C (2.2%), HLA-DRB1 (20.9%), HLA-DQA1 (23%), and HLA-DQB1 (28.4%). Because of that, we opted to consider only the direct calls for *HLA-A*, *HLA-B*, and *HLA-C*, and only the imputed alleles from *HLA-DRB1*, *HLA-DQA1*, and *HLA-DQB1*.

### Statistical analyses for association

The usual threshold for genome-wide significance in GWAS studies is *P*< 10^-8^, defined based on the average number of segregation blocks in European genomes. However, here, we are focusing on 2,346 SNPs across the MHC (not the full genome), in a different population (admixed Brazilians), and in a region with different LD patterns than the rest of the genome. Therefore, we calculated the number of different segregation blocks observed across our data by using Haploview and the confidence intervals algorithm (38). We detected exactly 100 segregation blocks with all bi-allelic markers with minimum allele frequencies of 2%. Therefore, we set alpha = 0.05/100 = 0.0005 as the threshold for detecting an associated variant despite multiple tests (the red lines in the Manhattan plots). We also report candidates that reach a 10-fold higher threshold, alpha < 0.005 (the blue line in the Manhattan plots) to avoid missing potential variants associated with Covid 19 severity.

We used plink2 to fit a logistic regression that considers each variable site, allotype, or amino acid residue as an independent marker. We created a plink-format table file containing a column for each allele of a SNP, every allotype, each amino acid in a specific position, and the dosage observed for the samples (from 0 to 2). The regression analysis, performed in R, considered sex and genetic ancestry as covariables in all comparisons. We did not include age as covariable to adjust *P*-values because of the super elderly. To evaluate the amino acid residues, we first aligned the predicted protein sequence of all individuals. While we considered all SNPs that have passed the filter described above, for the allotype and amino acid residues, we considered only the following genes: *HLA-A*, *HLA-B*, *HLA-C*, *HLA-E*, *HLA-F*, *HLA-G*, *MICA*, *MICB*, *HLA-DRA*, *HLA-DRB1*, *HLA-DQA1*, *HLA-DQB1*, *HLA-DPA1*, *HLA-DPB1*, *HLA-DOA*, *HLA-DOB*, *HLA-DMA*, *HLA-DMB*, *TAP1*, and *TAP2*.

### Modeling of HLA structures and analysis of immunogenic regions and spike epitopes

Aiming to enhance our understanding of underlying mechanisms for HLA alleles associated with disease severity and the mechanisms underlying the associations, we have predicted the HLA molecule structure and the SARS-CoV-2 peptides that can bind to these HLA versions. The detailed methods are in the supplementary material.

## Results

### Covid-19 demographic data

**Supplementary Table S1** presents demographic data and mean genome-wide genetic ancestry for each group. The mean age for the MILD group (66.9 years) is significantly higher than the SEVERE group (51.3 years), *p* < 10^-5^. There are more women in the MILD group (58.3%) and among the super elderly (74.7%) than in the SEVERE Covid-19 (43.6%).

All groups present similar proportions of genetic ancestry, except the SEVERE group which has a greater African and Native American ancestry than the MILD or the general elderly population from the same city (**Supplementary Table S1**). On one hand, ancestry might be related to Covid-19 severity, as observed for some Covid-19 comorbidities. For example, diabetes is a risk factor for severe Covid-19 (39) and is more frequent in individuals with higher African and Native-American ancestry in some populations (40). On the other hand, the lower socioeconomic status of a Brazilian citizen is correlated with a higher African and Native American ancestry. Most of the severe cases came from public hospitals, which, on average, are of poorer quality and lower efficiency, and then enriched for individuals with lower socioeconomic status (41). Nevertheless, the reported association results were controlled for genetic ancestry, which allowed adjustment to some extent for the socioeconomic scores.

### Extreme outcomes for Covid-19 severity

**Figure 2:**
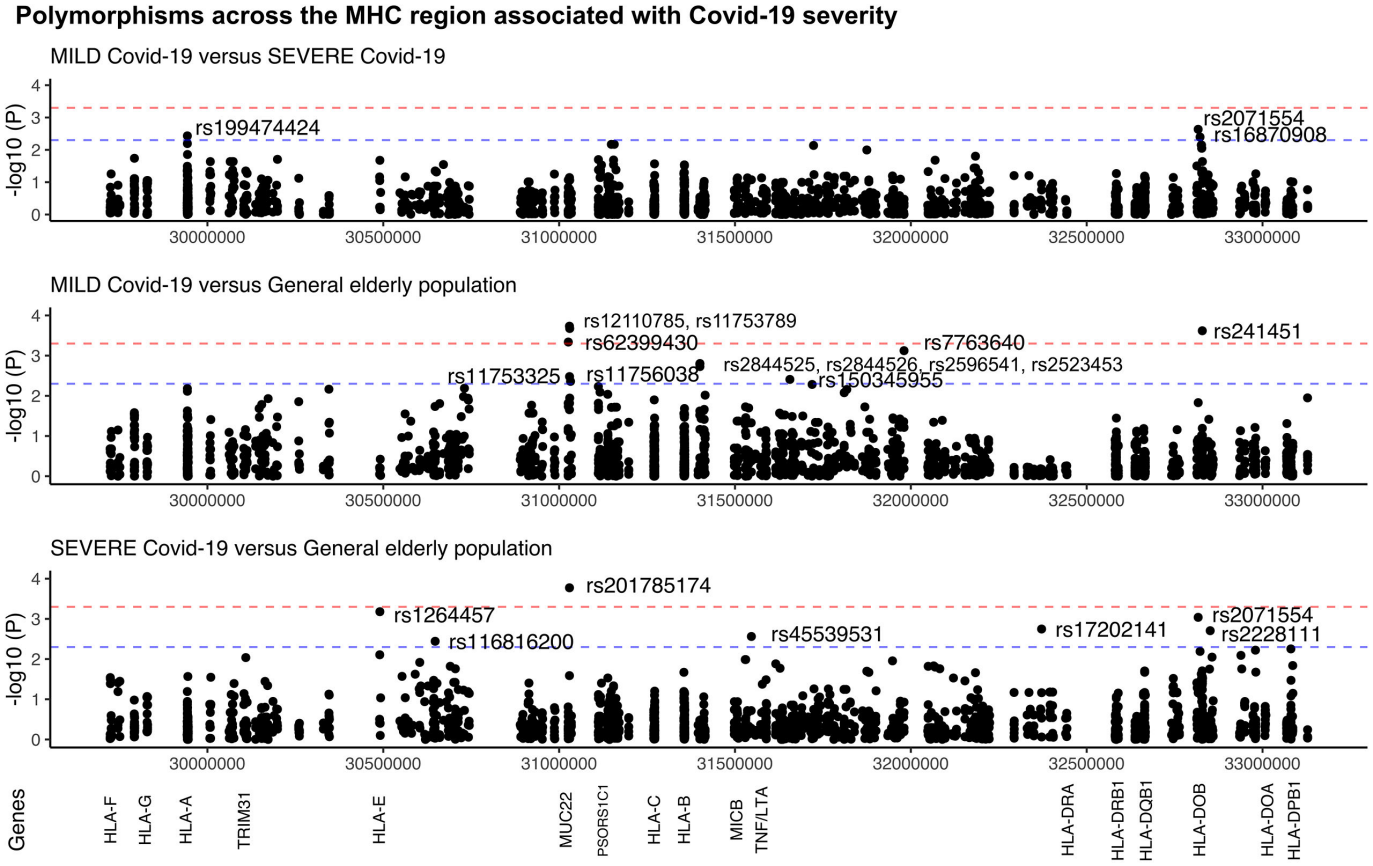
Manhattan plot illustrating differences among patients with MILD and SEVERE Covid-19, and the general elderly population from the same city. The general elderly population consists of whole-genomes (WGS) of 1,170 elders from São Paulo city, with unknown status regarding Covid-19. We considered only variants with a minor allele frequency of 1% in at least one of the groups, with no more than 5% of missing alleles in both groups, and with a ratio between read depth for each allele in heterozygous sites ranging from 0.3 to 0.5 for Exomes (the MILD and SEVERE groups). The red line marks the threshold for detecting a variant associated with the phenotype by calculating the number of segregation blocks observed in our data. The blue line marks the suggestive threshold for a candidate variant, *p* < 0.005. Most *MUC22* variants are in Linkage Disequilibrium (r^2^ > 0.8).

The comparison between the SEVERE and MILD groups revealed three missense candidate variants that are 2-3 times more frequent in the SEVERE group than in MILD, coinciding with genes *HLA-A*, *HLA-DOB*, and *TAP2* (**Figure 2** and **Table 1**). The *HLA-A* variant is also significantly less frequent in the MILD group than in the general elderly population (*p* = 0.0066), and *HLA-DOB* and *TAP2* variants are also significantly overrepresented in the SEVERE group compared to the general elderly population (*p* = 0.0009 and *p* = 0.0064, respectively), according with **Table 1**.

**Table 1:** Polymorphisms across the MHC region (SNPs and indels) that are associated with Covid-19 severity in Brazilian patients.

Position(Chr6, hg38)	SNP	Allele	Gene	LD group	Type	Severe (N = 55)	Mild (N = 163)	General population (N = 1.170)	Severe vs mild (p)	Severe vs general population (p)	Mild vs general population (p)
29942944	rs199474424	C	*HLA-A*	none	missense	0.2091	0.0890	0.1543	0.0037	0.1135	0.0066
30490287	rs1264457	G	*HLA-E*	none	missense	0.5545	0.4540	0.4325	0.0210	0.0007	0.6720
30647507	rs116816200	A	*C6orf136*	none	intronic	0.0545	0.0337	0.0175	0.2926	0.0036	0.1671
31026089	rs62399430	T	*MUC22*	A	missense	0.1273	0.2362	0.1449	0.0849	0.5381	0.0005*
31029301	rs11753789	C	*MUC22*	A	synonymous	0.1182	0.2393	0.1436	0.0720	0.4688	0.0002*
31029557	rs11756038	G	*MUC22*	A	missense	0.1182	0.2025	0.1299	0.1659	0.6488	0.0033
31030047	rs12110785	C	*MUC22*	none	missense	0.1636	0.2577	0.1778	0.0867	0.6170	0.0002*
31032362	rs11753325	T	*MUC22*	A	synonymous	0.1182	0.2025	0.1278	0.2361	0.7307	0.0044
31399930	rs2844526	C	*MICA*	B	upstream	0.4364	0.3712	0.4692	0.4112	0.6266	0.0016
31400061	rs2596541	A	*MICA*	B	upstream	0.4364	0.3712	0.4692	0.4113	0.6266	0.0016
31400348	rs2523453	G	*MICA*	B	upstream	0.4455	0.3742	0.4718	0.3335	0.7015	0.0019
31400377	rs2844525	C	*MICA*	B	upstream	0.4364	0.3712	0.4697	0.4112	0.5952	0.0016
31546626	rs45539531	A	*ATP6V1G2/DDX39B*	none	upstream	0.0727	0.0337	0.0282	0.0841	0.0027	0.7841
31656057	rs150345955	G	*APOM*	none	missense	0.0000	0.0184	0.0034	0.5197	0.9881	0.0039
31980631	rs7763640	C	*STK19/C4A*	none	intronic/upstream	0.0182	0.0123	0.0667	0.4561	0.1179	0.0008
32371721	rs17202141	G	*TSBP1*	none	downstream	0.0364	0.0031	0.0047	0.1123	0.0018	0.6683
32816899	rs2071554	T	*HLA-DOB*	none	missense	0.1364	0.0552	0.0607	0.0023	0.0009	0.7990
32822312	rs16870908	A	*TAP2*	none	missense	0.1364	0.0521	0.0697	0.0040	0.0064	0.5050
32851137	rs2228111	A	*TAP1*	none	missense	0.0364	0.0061	0.0056	0.2484	0.0020	0.3688

The SABE cohort represents the general population frequencies in the same city as the Covid-19 patients. *These variants achieved the threshold for a variant associated with mild Covid-19 infection. All others are candidate variants. LD (Linkage Disequilibrium) groups, A: r^2^ > 0.8, B: r^2^ > 0.98.

Probably because of the relatively small and different sample size of both the MILD and SEVERE groups, some variants are highlighted when compared to the general elderly population but not when comparing the Covid-19 groups, as, the susceptibility missense variants rs1264457 at *HLA-E* and rs2228111 at *TAP1*, among others. On the other hand, the protective missense variants at *MUC22* are significantly overrepresented among patients with MILD Covid-19 (**Figure 2** and **Table 1**).

We also evaluated whether variants are associated with Covid-19 severity in younger patients by removing the super elderly from the MILD group. The resulting smaller sample size did not allow the identification of candidate variants in this comparison. However, the pattern observed in **Table 1** was maintained.

We explored the allotypes and amino acid frequencies in different groups (**Table 2**). DOB*01:02 and the amino acid that defines this allotype, 18Q, are overrepresented in the SEVERE group compared to the MILD and the general elderly population. This amino acid exchange is related to the rs2071554 variant described in **Table 1**. HLA-E*01:03 and the main amino acid exchange that composes this allotype, 128G, is significantly overrepresented in the SEVERE group when compared to the general elderly population (*p* < 0.005) and the MILD group (*p* < 0.05). This amino acid exchange is related to rs1264457 (**Table 1**). All variants that define the allotype MICA*008 are overrepresented in the MILD group when compared to the general population (**Table 2**).

**Table 2:** MHC allotypes and amino acid residues associated with Covid-19 disease severity in Brazil.

Allotype	Severe covid-19 (*N* = 55)	Mild covid-19(*N* = 163)	General population (*N* = 1170)	Severe vs mild (*P*)	Severe vs general population (*P*)	Mild vs general population (*P*)
DOB*01:02 or 18Q	0.1364	0.0552	0.0607	0.0005	0.0007	0.7951
E*01:03	0.5455	0.4356	0.4137	0.0173	0.0001	0.7079
HLA-E^G^	0.5545	0.4324	0.4539	0.0227	0.0008	0.7176
MICA*008	0.2727	0.3221	0.2436	0.1758	0.6026	0.0039
Amino Acid exchanges that define MICA*008	0.2727	0.3220	0.2444	0.1759	0.6332	0.0044
HLA-A 62R/63N	0.2090	0.0889	0.1542	0.0017	0.1345	0.0055
HLA-A 156W	0.1636	0.0797	0.1380	0.0254	0.3280	0.0027
TAP1 286F	0.0363	0.0061	0.0055	0.2133	0.0001	0.4647

*adjusted for sex and genetic ancestry.

Two amino acid residues at HLA-A, 86R and 87N (full-length protein) or 62R and 63N (mature protein), are significantly overrepresented in the SEVERE group when compared to the MILD group (*p* < 0.005), and overrepresented in the general elderly population when compared to the MILD group (*p* < 0.01). Likewise, HLA-A residue 156/W is less frequent in the MILD group compared to the SEVERE (*p* < 0.05) and the general elderly population (*p* < 0.005). Residues 62R and 63N are associated with many HLA-A allotypes, including A*25, A*26, A*33, A*34, A*66, A*68, and A*69. Residue 156/W is associated with allotypes A*25, A*26, A*34, A*43, A*66, and A*68.

The linkage disequilibrium (LD) pattern in **Figure S7** indicates that most Covid-19-associated *MUC22* polymorphisms are in strong LD. Likewise, the MICA variants (all associated with MICA*008) are in strong LD. *MUC22* and *MICA* are independent signals. The signals from *HLA-DOB* and TAP2 might not be independent. There is no LD between the *HLA-A* variant and other relevant variants across the MHC.

### Super elderly patients recovered from Covid-19 as compared to the elderly general population

**Figure 3:**
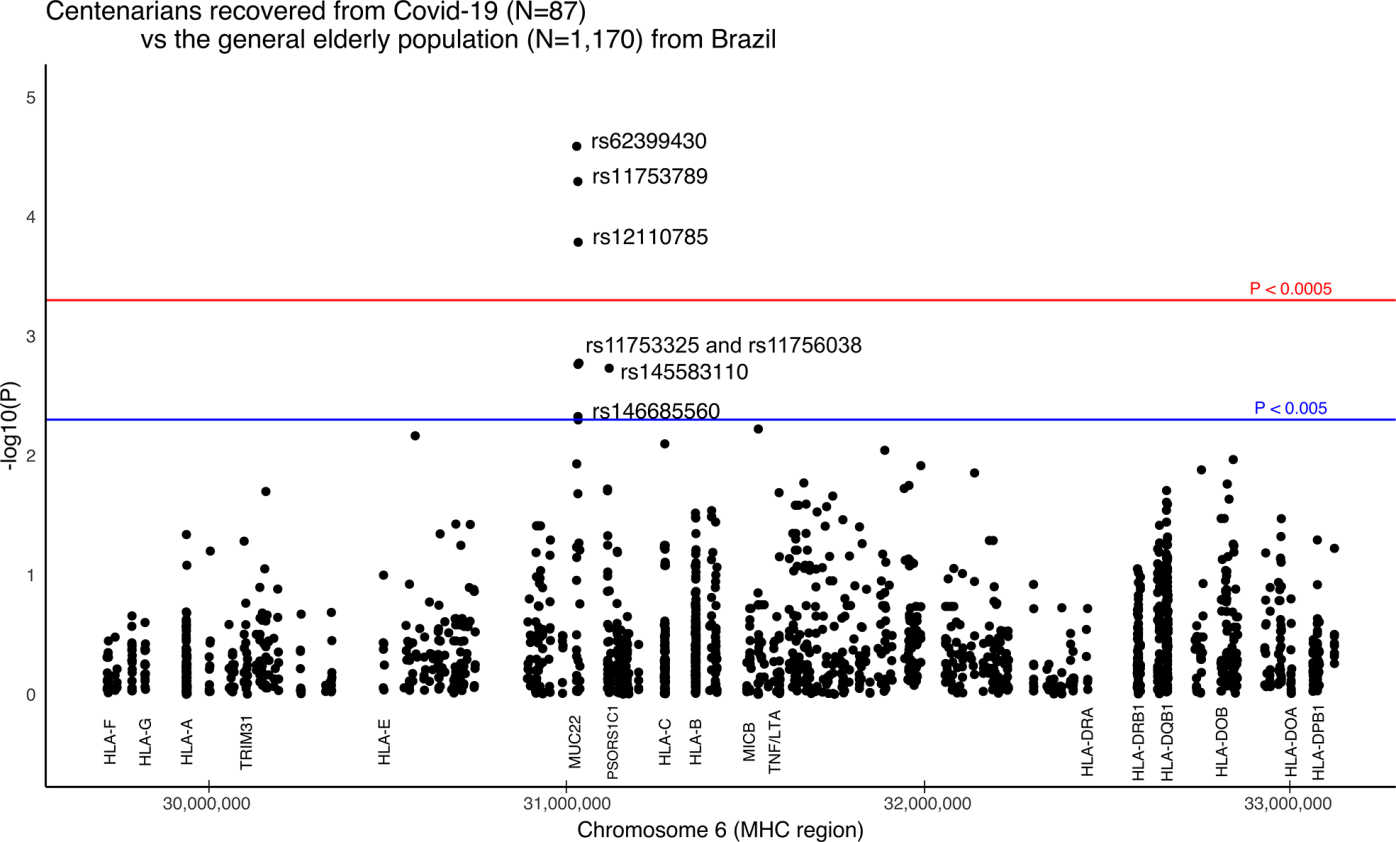
Manhattan plot illustrating differences between Brazilian centenarians (>90 y) recovered from Covid-19 and the general elderly population. The general population consists of whole-genomes (WGS) of 1,170 elders from São Paulo city (> 65 y), with unknown status regarding Covid-19. The centenarian group includes exomes of 87 patients more than 90 years old that presented mild Covid-19 symptoms. We considered only variants with a minor allele frequency of 1% in at least one of the groups, with no more than 5% of missing alleles in both groups, and with a ratio between read depth for each allele in heterozygous sites ranging from 0.3 to 0.5 for Exomes. The red line marks the threshold for detecting a variant associated with the phenotype by calculating the number of segregation blocks observed in our data. The blue line marks the suggestive threshold for a candidate variant, *p* < 0.005. Most *MUC22* variants are in Linkage Disequilibrium (r^2^ > 0.8).

The strongest signal coincides with gene *MUC22*, rs62399430, two times more frequent among the super elderly than in the SEVERE group (*p* = 0.0057, OR=0.30). Moreover, when super elderly are compared to the general elderly population from the same city, we detected candidate variants in two genes, *MUC22 and PSORS1C1/CDSN* (**Figure 3**). Most of the signals coincide with gene *MUC22*. Variants rs62399430, rs11753789, and rs12110785 (*p* < 0.0002, OR > 2.0) are, in general, two times more frequent among super elderly recovered from Covid-19. Two of these are missense variants. Most *MUC22* variants are in Linkage Disequilibrium (r^2^> 0.8, **Figure S7**). These variants are also overrepresented in the MILD Covid-19 group, which includes most of the super elderly patients and younger patients with mild Covid-19. Another candidate variant for protection is rs145583110, an intronic variant from *PSORS1C1* or exonic for *CDSN*, which is 3 times more frequent among the super elderly than in the general population (*p* = 0.0018, OR = 3.85).

The allotype and amino acid residue frequencies reveal no relevant association when comparing super elderly with MILD Covid-19 and younger patients with SEVERE Covid-19, or when super elderly are compared to the general elderly population.

## Discussion

Here we investigated the polymorphisms across the MHC region associated with Covid-19 disease severity in patients with extreme phenotypes: young adults with severe Covid-19, and super elderly individuals with mild Covid-19. All the samples were collected between June and October 2020, before new SARS-CoV-2 variants were reported in Brazil (especially Gamma) and before the onset of the Brazilian vaccination program against Covid-19. This data may shed some light on the mechanisms underlying SARS-CoV-2 resistance, particularly for the earlier SARS-CoV-2 strains in unvaccinated individuals. All samples from the control elderly Brazilian population were collected before the SARS-CoV-2 outbreak.

We applied a bioinformatics pipeline to correct alignments and call reliable genotypes and HLA alleles and detected some associated and candidate variants that influence infection severity, some related to genes from the antigen presentation pathway and others from different pathways. This section will focus on the strongest hits and the frequent candidate variants associated with the phenotypes, particularly *MUC22*, *HLA-A*, and *HLA-DOB*.

### *MUC22* variants and mild Covid-19

We detected missense *MUC22* variants associated with mild Covid-19 when evaluating all patients with mild symptoms, and the super elderly recovered from Covid-19. MUC genes encode mucins, and 16 different mucins have been identified in the lung (42). Mucins are high-molecular-weight glycoproteins that can be secreted or anchored to the cell membrane (transmembrane mucins) (42). *MUC22* is a member of the mucins’ family. It encodes transmembrane mucins expressed in the bronchi of the lungs and participates in the inflammatory and innate immune response (43, 44). Airway mucus comprises water, antimicrobial proteins, serum protein transudates, and mucus glycoproteins. It protects and lubricates the respiratory tract. However, excessive mucus production is related to inflammatory lung diseases (45), which are found in severe cases of Covid-19. Overexpression of MUC1 and MUC5AC mucins, for instance, play a key role in Covid-19 symptoms and may contribute to the high viscosity of airway mucus, leading to airflow obstruction and respiratory distress (46).

*MUC22* is up-regulated in infections with respiratory syncytial vírus (47). Some studies have been pointing to mucin´s role in Covid-19 (48, 49). The outcome of SARS-CoV-2 infection may be correlated with a signature of shed mucins in circulation from infected lung or respiratory tract epithelial cells (48). *MUC22* polymorphisms have been associated with diffuse panbronchiolitis (43) and asthma in Latinos (50).

One may argue that these variants are related to longevity and not with protection against severe Covid-19. However, this variant is not correlated with longevity because (a) the SABE sample refers to a census-based cohort of elderly Brazilians with an average age of 75, and thus variant associated with longevity would already be more frequent, and (b) their frequency in SABE is very similar to frequencies observed in European and Latin American populations (https://www.ncbi.nlm.nih.gov/snp/rs62399430#frequency_tab).

Except for rs146685560, all *MUC22* protective variants are correlated with higher expression of miR-6891 in many tissues, including the esophagus and lung (gtexportal.org). miR-6891 is co-expressed with *MUC22* in the alveolus (pneumocyte type II). Interestingly, miR-6891 is encoded in the MHC, and targets the ORF3a gene from SARS-CoV-2 (51), which encodes a sodium or calcium ion channel protein involved in replication and pathogenesis (52). Most importantly, miR-6891-5p is upregulated in Calu3 cells infected with SARS-CoV-2 (53). During the early stages of SARS-CoV-2 infection, ORF3a directs the host’s immune response (54), may induce lysosomal evasion (55, 56) and could promote cytokine storms by activating the NF-kB signaling and NLRP3 inflammasomes pathways (57). Therefore, we can hypothesize that a higher expression of miR-6891-5p, associated with all *MUC22* protective variants, may contribute to less severe symptoms during SARS-CoV-2 infection.

One possible explanation for higher miR-6891-5p expression is linkage with *HLA-B* since the *MIR6891* gene coincides with an *HLA-B* intron. In the present study, there is a clear association between the protective variant rs62399430/T and some *HLA-B* alleles, such as B*35:01, B*35:03, B*48:02, and B*51:01. All of these *HLA-B* alleles are listed as high mRNA expressing alleles (58–60). Thus, higher *HLA-B* mRNA expression might be correlated with higher miR-6891 expression, and *MUC22* variants are tagging this phenotype.

Also, *MUC22* is approximately 100 kb from *CCHCR1* gene, the most important signal in the MHC region for Covid-19 susceptibility, according to the Covid-19 Host Genetics Consortium (Covid19hg - https://app.covid19hg.org/). All *CCHCR1* variants associated with Covid-19 (29, 61) are intronic and were not captured by our genotyping method (whole-exome sequencing). The exomic *CCHCR1* variants included in our survey do not correlate with Covid-19 severity. This is expected since the meta-analysis provided by Covid19hg indicates that *CCHCR1* is associated with Covid-19 susceptibility but not with severity when comparing hospitalized and non-hospitalized patients (as performed here). Moreover, studying LD among the most relevant variants from *MUC22* and *CCHCR1*, we detected a weak LD (r2 < 0.3, D’ < 0.7). Covid19hg effort detected no relevant signal from *MUC22*, including the SNPs described here. Although our *MUC22* findings are not cross-validated by the Covid19hg, we must consider that we are evaluating a very different cohort, aged and super elderly individuals with mild Covid-19 and younger patients with severe Covid-19. The signal from *MUC22* is much stronger in the super elderly group. The frequency of rs62399430 among the super elderly with mild Covid-19 is 2x higher than the observed among Europeans and in the general elderly populations and 3.8x higher than in Africa. Therefore, considering study designs and cohort ancestries, the *MUC22* signal might be ancestry-specific and independent of *CCHCR1*.

### HLA-DOB*01:02/rs2071554 and severe Covid-19

The HLA-DO molecule is a heterodimer formed by two heavy chains, HLA-DOA and HLA-DOB. HLA-DO is a non-classical MHC-II molecule that does not present peptides on the cell surface, but it is required to efficiently load endosomal peptides onto MHC-II molecules (62–66). Thus, modifications in *HLA-DOB* may directly influence antigen presentation in the MHC class II pathway.

The HLA-DOB*01:02 allele has also been associated with an increased risk of death in patients with non-small cell lung cancer; reduced median survival time (67, 68), with type 1 diabetes (69), which is a major comorbidity related to severe Covid-19 (39), and with resistance to SARs-CoV-2 infection (22). DOB*01:02 has a different signal peptide, which may influence cellular localization and trafficking of the protein (15), possibly leading to inadequate antigen presentation.

HLA-DOB*01:02 is rare (0.6%) among SARs-CoV-2 resistant individuals (22), has similar frequencies of around 6% among patients with mild Covid-19 or the control elderly population, and reaches 13.6% in patients with severe Covid-19 (**Table 1**). The frequency of DOB*01:02 among the super elderly is similar to the general elderly population.

DOB*01:02 is frequent in Africa (reaching the same frequency as the SEVERE group) and less frequent in Europe. Although the percentage of Native-American and African ancestries are much higher in the SEVERE group than others, the associations described here are adjusted for genetic ancestry and sex. Because DOB*01:02 frequencies vary among different biogeographic regions, other studies are unlikely to find similar results unless addressing African or admixed populations comparable to the Brazilian one.

### *HLA-A*, *TAP1*, and *TAP2* polymorphisms and severe Covid-19

We detected three different associations involving genes from the class I antigen presentation pathway. These associations involve *TAP1* and *TAP2*, which participate in the peptide pumping from the cytoplasm to the endoplasmic reticulum, and gene *HLA-A*, which will present these peptides on the cell surface to T CD8 lymphocytes (**Tables 1**, **2**). High expression levels of TAP1 and TAP2 are correlated with the amount of virus in lung tissue (70). The *TAP2* signal might be a hitchhiking association due to linkage with HLA-DOB*01:02 (**Figure S7**).

For HLA-A, the amino acid residues at positions 62 and 63 (mature protein) depend on the haplotype formed by four different variants, rs1059455, rs1064588, rs2230991, and rs199474424. Their presence is associated with severe Covid-19. The fact that (a) this combination of amino acids only occurs when there is a specific haplotype, (b) this haplotype occurs in many different *HLA-A* alleles, and (c) allele frequencies vary in different populations, may explain why we did not detect any association between *HLA-A* allotypes with Covid-19 severity and why previous surveys described different results or no association (23, 71–77).

One possible mechanism for these associations is different binding affinities to SARs-CoV-2 peptides coupled with the subset of peptides pumped by TAP. The higher frequency of 62R-63N in the SEVERE group is related to alleles A*33:03, A*68:01, and A*68:02. A*68 alleles are among the strong binders for SARs-CoV-2 peptides (78, 79) and among the best binders for respiratory viruses (70).

To investigate whether residues 62R/63N could be interfering with the subset of antigens presented by *HLA-A*, we performed an *in silico* prediction of the antigen processing pathway impact on the presentation ability of the alleles carrying 62R/63N. Since the set of MHC alleles that an individual presents defines the ligandome on its cell surface, we predicted the peptidome of inspected alleles and compared them with the corresponding immunogenic regions from the SARS-CoV-2 spike protein. The comparison did not provide evidence that the investigated alleles lack the potential to present T cell epitopes already described for SARS-CoV-2 sequences (**Supplementary Figures S3**, **S4**). Additionally, we extracted the frequency of response for each predicted peptide from each 62R/63N sample alleles to compare their average values of immunogenicity frequencies with other alleles associated with good and bad outcomes in Covid-19. Again, the alleles carrying 62R/63N and overrepresented in the severe group exhibited similar numbers, indicating that the presentation ability was not responsible for the impaired response (**Supplementary Figure S5**).

To infer if the mutations could be interfering with immunogenicity triggering, we also looked for alterations present in the 62R/63N alleles that could impact the TCR interaction surface of the MHC cleft. The HLA structural models were screened, looking for shared features in regions usually contacted by complementarity-determining region 3 (CDR3) loops (**Supplementary Figure S6**). Hierarchical clustering analysis showed that four out of eight investigated alleles presented an electrostatic potential distribution fingerprint in the probed area. This physicochemical element was already described as pivotal to cytotoxicity elicitation, as described previously (80).

**Figure 4:**
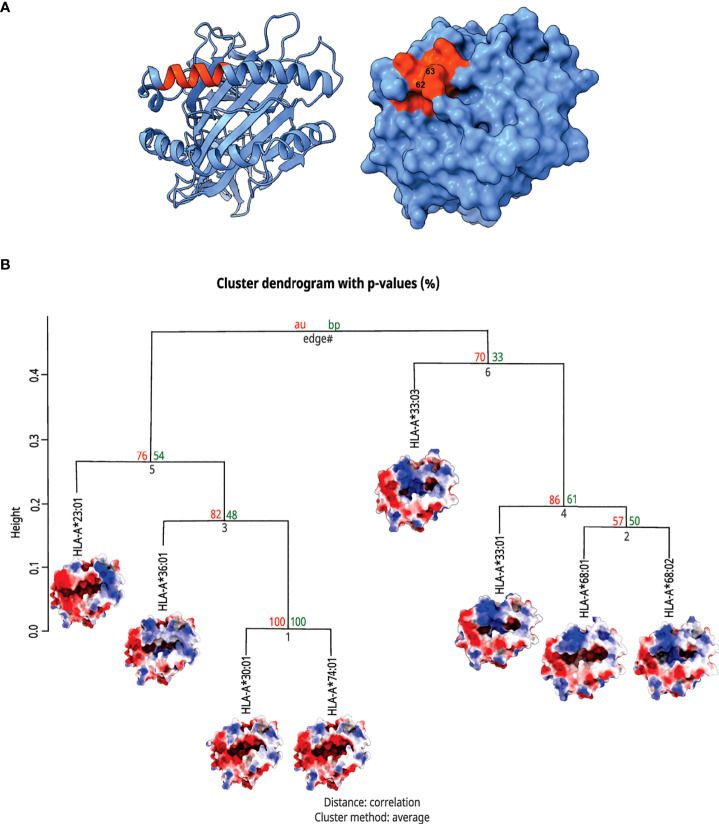
Top view of HLA-A molecules and hierarchical clusterization of HLA-A alleles. In **(A)** there is a MHC structure in both the ribbon and surface visualizations from the region that interacts with the TCR, respectively. The area in orange represents the zone proximal about the residues 62 and 63. In **(B)** we can see the cluster dendrogram of the analyzed MHCs. Under each allele name, there is the surface image that was generated for electrostatic potential distribution. In the right branch, we can see the cluster of alleles carrying rs199474424 (please refer to **Figure 2**), HLA-A*33:01, A*33:03, A*68:01, and A*68:02, with their respective shared molecular fingerprint.

Interestingly, the clustered alleles were HLA-A*33:01, A*33:03, A*68:01, and A*68:02, the ones with higher frequencies among patients with Severe Covid-19 (**Figure 4**). These analyses emphasize the need for a deeper investigation when we are dealing with HLA alleles and their involvement with immunogenic issues. Only looking for ligandome predictions can underestimate the whole importance of these structures in T cell stimulation.

Among the alleles with increased frequency in the SEVERE group, A*68:02 is particularly frequent in Africa, A*62:01 among Native Americans, and A*33:03 in Asia (www.allelefrequencies.net). Although the SEVERE group from Brazil presents a higher African and Native American ancestry than the MILD group (**Supplementary Table S1**), the associations presented here (62R/63N, *p* = 0.0017, OR=2.7) are adjusted for ancestry and sex. This *p*-value is much lower, 0.0003, and significant even after correction for multiple tests within the *HLA-A* locus when not adjusted for ancestry. Therefore, population stratification should be ruled out as the main issue leading to these results. Since the associated alleles are most frequent in non-European populations, particularly Native Americans and Africans, it is unlikely that any study addressing European ancestry samples (23, 25, 74, 75, 77, 81–83) would find similar results. In fact, most of the previous studies evaluate European patients and find different results than the one presented here. Nevertheless, a frequency analysis of HLA alleles among Covid-19 infected patients from Saudi Arabia found HLA-A*68 among the most common alleles associated with severe disease (84), and A*68 is relatively common in Saudi Arabia (www.allelefrequencies.net). These results demonstrate the importance of addressing admixed populations such as Brazilians and other less-studied population samples.

## Conclusions

In short, here, we performed an in-depth analysis of the MHC region in a cohort of unvaccinated super elderly individuals with Covid-19 that presented mild, or no symptoms compared with a group of younger patients with severe Covid-19 and/or a lethal outcome. We used a method to call genotypes and haplotypes in the MHC that minimizes alignment and genotyping errors. Interestingly, the strongest signals in the MHC region for candidate variants protecting against severe Covid-19 coincide with gene *MUC22*. Missense variants at *MUC22* are more frequent among the super elderly and in the MILD group than in the SEVERE group and the general elderly population. We hypothesized that *MUC22* might play an important protective role against severe Covid-19. Functional studies must be placed to evaluate the true impact of such variants on *MUC22* function.

## Data availability statement

The datasets presented in this study can be found in online repositories. The names of the repository/repositories and accession number(s) can be found below: European Genome-phenome Archive (EGA), under accession number EGAS0000100637.

## Ethics statement

This study was approved by the Committee for Ethics in Research of the Institute of Biosciences at the University of São Paulo (CAAE 34786620.2.0000.5464). The patients/participants provided their written informed consent to participate in this study.

## Author contributions

EC, MC, MN, and MZ contributed to the conceptualization. MC, LM, and MVRS contributed to data curation. EC, MN, MOS, NS, KN, IO, EA, ES, and GV contributed to the formal analysis. MZ contributed to funding acquisition. MC, MN, MOS, EC-N, and KS contributed to the investigation. EC, MN, MOS, and KN contributed to the methodology. MZ contributed to the project administration. EC, MN, and MOS contributed the software. EC, MN, MOS, and KN contributed to visualization. EC, MZ, MC, MN, KS, and MOS contributed to writing–original draft. NS, RP, VAOC, CC, CM-J, GV, DM, LM, MVRS, JW, JE, VRC, JM, EN, JK, RB, MH, LD’A, AR-F, PB, AD, MD, PS, MP-B, and MZ contributed to writing–review, and editing. All authors contributed to the article and approved the submitted version.

## Funding

This work was supported by the São Paulo Research Foundation (FAPESP/Brazil) (grant numbers 2013/08028-1, 2014/50931-3, 2019/19998-8, and 2020/09702-1), the National Council for Scientific and Technological Development (CNPq) (grant number 465355/2014-5), and JBS S.A. (grant number 69004). FAPESP/Brazil (Grant numbers 2013/17084-0 and 2017/19223-0) and the United States National Institutes of Health (NIH) (R01 GM075091) supported the development of the HLA and KIR pipeline and the genetic ancestry approach. This study was also supported by the Coordenação de Aperfeiçoamento de Pessoal de Nível Superior-Brasil (CAPES)-Finance Code 001 and Fleury Group (Project NP-565). The funders were not involved in the study design, collection, analysis, interpretation of data, the writing of this article, or the decision to submit it for publication.

## Acknowledgments

The authors are extremely grateful to all volunteers for their participation and collaboration, the nurses for sample collection, the technical team for the material process and data analysis, and the Fleury Laboratory for serology tests. Special thanks to Brazilian Senator Mara Gabrilli for financial support and to JBS S.A. for the additional funding. The funders were not involved in the study design, collection, analysis, interpretation of data, the writing of this article, or the decision to submit it for publication.

## Conflict of interest

The authors declare that the research was conducted in the absence of any commercial or financial relationships that could be construed as a potential conflict of interest.

## Publisher’s note

All claims expressed in this article are solely those of the authors and do not necessarily represent those of their affiliated organizations, or those of the publisher, the editors and the reviewers. Any product that may be evaluated in this article, or claim that may be made by its manufacturer, is not guaranteed or endorsed by the publisher.
