# Supplementary material for: MUC22, HLA-A, and HLA-DOB variants and COVID-19 in resilient super-agers from Brazil
Source: Front Immunol. 2022 Oct 25;13:975918. doi: 10.3389/fimmu.2022.975918 (PMC9641602; doi:10.3389/fimmu.2022.975918)
Supplement: Supplementary file 1 [file DataSheet_1.docx]

***Supplementary Material***

**MUC22, HLA-A, and HLA-DOB variants and COVID-19 in resilient super-agers from Brazil**

**Erick C. Castelli^1,2*+^, Mateus V. de Castro^3*+^, Michel S. Naslavsky^3,4^, Marilia O. Scliar^3^, Nayane S. B. Silva^2^, Raphaela N. Pereira^2^, Viviane A. O. Ciriaco^2^, Camila F. B. Castro^2,5^, Celso T. Mendes-Junior^6^, Etiele de S. Silveira^7^, Iuri M. de Oliveira^7^, Eduardo C. Antonio^7^, Gustavo F. Vieira^7,8^, Diogo Meyer^4^, Kelly Nunes^4^, Larissa R. B. Matos^3^, Monize V. R. Silva^3^, Jaqueline Y. T. Wang^3^, Joyce Esposito^3^, Vivian R. Cória^3^, Jhosiene Y. Magawa^9,10,11^, Keity S. Santos^9,10,11^, Edecio Cunha-Neto^9,10,11^, Jorge Kalil^9,10,11^, Raul H. Bortolin^12^, Mario H. Hirata^12^, Luiz P. Dell’Aquila^13^, Alvaro Razuk-Filho^13^, Pedro B. Batista Junior^13^, Amaro N. Duarte Neto^14^, Marisa Dolhnikoff^14^, Paulo H. N. Saldiva^14^, Maria Rita Passos-Bueno^3,4^,and Mayana Zatz^3,4*^**

^1^ Department of Pathology, School of Medicine, São Paulo State University (UNESP), Botucatu, Brazil;

^2^ Molecular Genetics and Bioinformatics Laboratory Experimental Research Unit (Unipex), School of Medicine, São Paulo State University (UNESP), Botucatu, Brazil;

^3^ Human Genome and Stem Cell Research Center, University of São Paulo, São Paulo, Brazil;

^4^ Department of Genetics and Evolutionary Biology, Biosciences Institute, University of São Paulo, São Paulo, Brazil;

^5^ Centro Universitário Sudoeste Paulista, Avaré, Brazil;

^6^ Departamento de Química, Faculdade de Filosofa, Ciências e Letras de Ribeirão Preto, Universidade de São Paulo, Ribeirão Preto, Brazil;

^7^ Programa de Pós-Graduação em Genética e Biologia Molecular - UFRGS – Brazil;

^8^ Laboratório de Saúde Humana In Silico - Programa de Pós-Graduação em Saúde e Desenvolvimento Humano - Universidade La Salle Canoas – Brazil;

^9^Departamento de Clínica Médica, Disciplina de Alergia e Imunologia Clínica, Faculdade de Medicina da Universidade de São Paulo, São Paulo, Brazil;

^10^Laboratório de Imunologia, Instituto do Coração (InCor), LIM19, Hospital das Clínicas da Faculdade de Medicina da Universidade de São Paulo, (HCFMUSP), São Paulo, Brazil;

^11^Instituto de Investigação em Imunologia - Instituto Nacional de Ciências e Tecnologia-iii-INCT, São Paulo, Brazil;

^12^Department of Clinical and Toxicological Analyses, School of Pharmaceutical Sciences, University of São Paulo, São Paulo, Brazil;

^13^Prevent Senior Institute, São Paulo, Brazil;

^14^Department of Pathology, School of Medicine, University of Sao Paulo, Sao Paulo, Brazil.

+ These authors contributed equally

*** Correspondence**: Mayana Zatz, mayazatz@usp.br; Erick C. Castelli, erick.castelli@unesp.br; Mateus V. de Castro, mateusvidigal@hotmail.com

# Supplementary Data

**Modeling of HLA structures**

The MHC alleles sequences in FASTA format were downloaded from IPD-IMGT/HLA Database [1]. The information about IPD Access and their respective alleles are in **Supplementary Table S2**. The sequences used for modeling the structures were HLA-A*23:01, HLA-A*30:01, HLA-A*33:03, HLA-A*36:01, HLA-A*68:01, HLA-A*68:02 e HLA-A*74:01. Information from Deb et al. 2022 was considered to choose alleles with a tendency for poor prognosis for Covid-19 (HLA-A*01:01 and HLA-A*30:02) and alleles for better prognosis (HLA-A*02:01, HLA-A*03:01, and HLA-A *11:01).

The sequences in FASTA format were used for the 3-D structure modeling with the Phyre2 tool Protein Homology/analogy Recognition Engine V 2.0 program [2]. The option of One-to-one threading was utilized in the Expert Mode. The crystal structure of the HLA-A*02 MHC allele (access number: 2V2W) downloaded from RCSB PDB (<https://www.rcsb.org/>) was used as the model structure. The quality of the generated model was evaluated with PROCHECK software in the PDBSum, disponible in EBI –European Molecular Biology- site (<https://www.ebi.ac.uk/>).

**Analysis of immunogenic regions and epitopes of MHC-I of the glycoprotein Spike (PD0CT2).**

The Immunome Browser tool responsible for Immune Epitope Database and Analysis Resource –IEDB ([www.iedb.org](http://www.iedb.org)) was used to recover the regions with experimentally described immunogenicity of the SARS-CoV-2 spike protein. The following parameters were used in the search: Assay: T cell, Epitope Source: Organism = SARS-CoV-2 (ID2697049), Antigen= Spyke Glycoprotein P0DTC2, MHC Restriction = Class I, Host = Human. The frequency graphs generated were used to identify the peptide position of the alleles in **Supplementary** **Table S2** that were found with the IEDB MHC-I Processing Predictions and plotted in **Supplementary Figure S3** and **Figure S4**. The output of the Immunome Browser is found in **Supplementary Table S3**.

The Proteassomal cleavage/TAP transport/MHC class I combined predictor (<http://tools.iedb.org/processing/>) was used to predict the presented HLA-I peptides for each investigated alleles in **Supplementary** **Table S2**. The SARS-CoV-2 spike (P0DTC2) sequence in FASTA format was used as input; the alleles of **Supplementary** **Table S1** were selected and a length of 9 amino acids was used. Other fields were kept default. The ten peptides with the highest Total Score were selected (**Supplementary Table S4** and **S5**). The information about the initial and final position of each peptide for each allele was considered to make the proper positioning in the image created with the Immuno Browser and can be verified in **Supplementary Figures S3** and **S4**.

To infer the response frequency for each of the ten peptides by MHC-I Processing Predictions, the Response Frequency (Response Freq) and Confidence Interval (CI) were extracted from Immunome Browser - IEDB. The Response Freq is the average overall epitopes mapped to that position and calculated as the number of positively responded subjects relative to the total number of those tested [3]. Statistical analyses of the data were performed using one-way analysis of variance (ANOVA) followed by Tukey’s multiple comparison test. p < 0.05 was considered signiﬁcant. Data were expressed as Response Freq. (95% CI) individual values, mean ± standard deviation (SD). Graph Pad Prism 8.0 software was used for the statistical analysis (GraphPad Inc., San Diego, CA).

**Electrostatic surface analysis**

Surface analyzes were performed using the ChimeraX software [4]. The molecular surfaces of the MHC alleles were added and colored later using files containing the calculation of electrostatic potential distributed on the desired molecular surface. These files containing the electrostatic surface calculations were generated using the Delphi software [5] which makes use of Poisson-Boltzmann equations to map the electrostatic content on a desired molecular surface. Having generated the surface images with the color indicating the distribution of electrostatic charges, a series of areas were selected to be analyzed by the ImageJ program [6]. The areas analyzed were chosen based on a previous work by Mendes *et al* [7], where the areas that had the highest contact rate with the TCR were selected for analysis; only those areas that would be in contact with the epitope carried by the TCR were removed. For further analysis, we utilized packages available in R studio to perform hierarchical clusterization to properly segregate different structures based on their surface color pattern.

**References:**

1. Robinson J, Barker DJ, Georgiou X, Cooper MA, Flicek P, MarshSGE. 2020. The IPD-IMGT/HLA Database. Nucleic AcidS Research. 43:D948-D955.
2. Kelley, L., Mezulis, S., Yates, C. *et al.* The Phyre2 web portal for protein modeling, prediction and analysis. *Nat Protoc* **10,** 845–858 (2015).<https://doi.org/10.1038/nprot.2015.053>
3. Vita R, Mahajan S, Overton JA, Dhanda SK, Martini S, Cantrell JR, Wheeler DK, Sette A, Peters B. The Immune Epitope Database (IEDB): 2018 update. Nucleic Acids Res. 2018 Oct 24. doi: 10.1093/nar/gky1006. [Epub ahead of print] PubMed PMID: [30357391](https://www.ncbi.nlm.nih.gov/pubmed/30357391).
4. [UCSF ChimeraX: Meeting modern challenges in visualization and analysis.](https://www.ncbi.nlm.nih.gov/pubmed/28710774) Goddard TD, Huang CC, Meng EC, Pettersen EF, Couch GS, Morris JH, Ferrin TE. *Protein Sci.* 2018 Jan;27(1):14-25.
5. Li, L., Li, C., Sarkar, S. *et al.* DelPhi: a comprehensive suite for DelPhi software and associated resources. *BMC Biophys* **5,** 9 (2012). https://doi.org/10.1186/2046-1682-5-9
6. Schneider, C. A., Rasband, W. S., & Eliceiri, K. W. (2012). NIH Image to ImageJ: 25 years of image analysis. *Nature Methods*, *9*(7), 671–675. [doi:10.1038/nmeth.2089](https://doi.org/10.1038/nmeth.2089)
7. Mendes MF, Antunes DA, Rigo MM, Sinigaglia M, Vieira GF. Improved structural method for T-cell cross-reactivity prediction. Mol Immunol. 2015 Oct;67(2 Pt B):303-10. doi: 10.1016/j.molimm.2015.06.017. Epub 2015 Jul 2. PMID: 26141239.

# Supplementary Figures and Tables

## Supplementary Figures


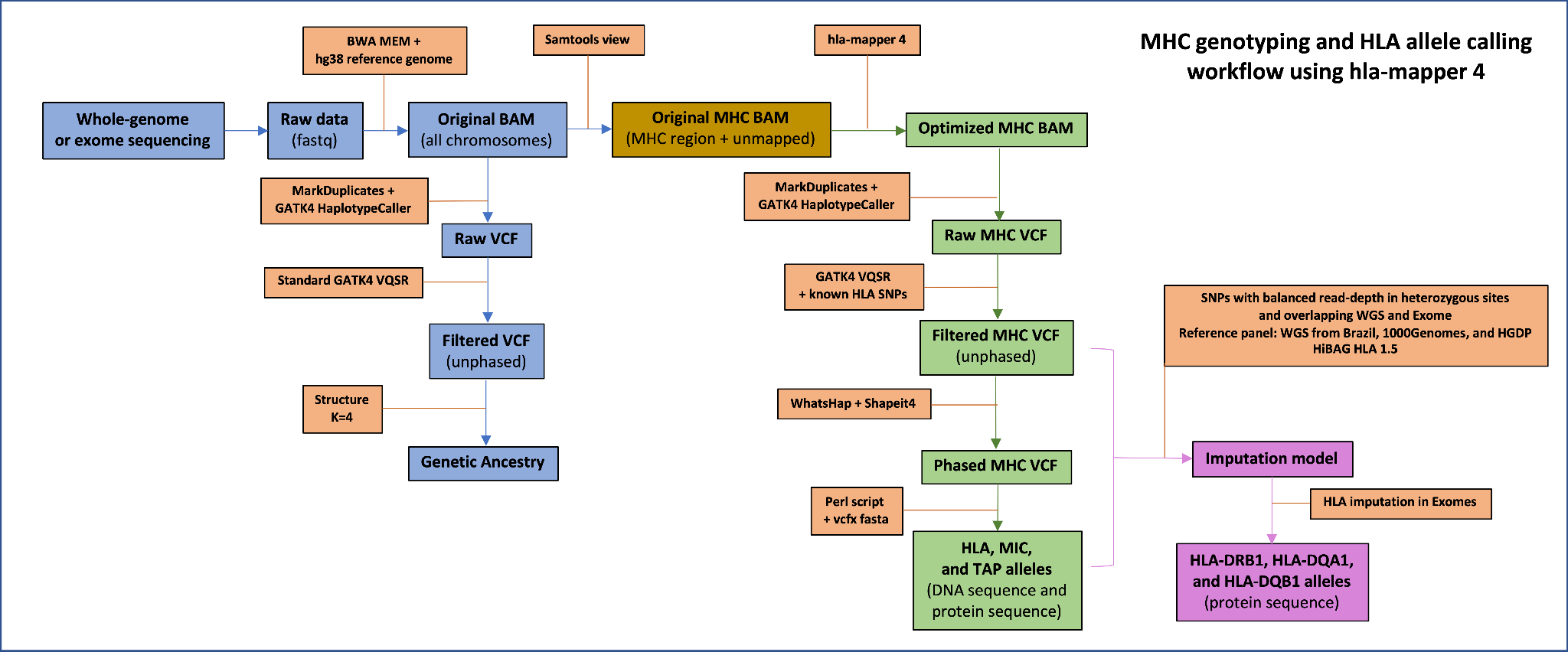


**Supplementary Figure S1:** MHC genotyping and HLA allele calling workflow using HLA-mapper 4. The same workflow was applied to whole-genome sequencing and Exome sequencing with minor modifications. In orange, the methods and algorithms used in each step. In blue, the standard workflow for processing raw sequence data to call SNPs in the entire human genome. In green, the hla-mapper 4 workflow to optimize read alignment, call genotypes, and haplotypes for MHC genes. In pink, the imputation workflow we used for *HLA-DRB1*, *HLA-DQA1*, and *HLA-DQB1*. The reference panel for imputation considers 1,171 Brazilian samples and all samples from the 1000Genomes and HGDP datasets.


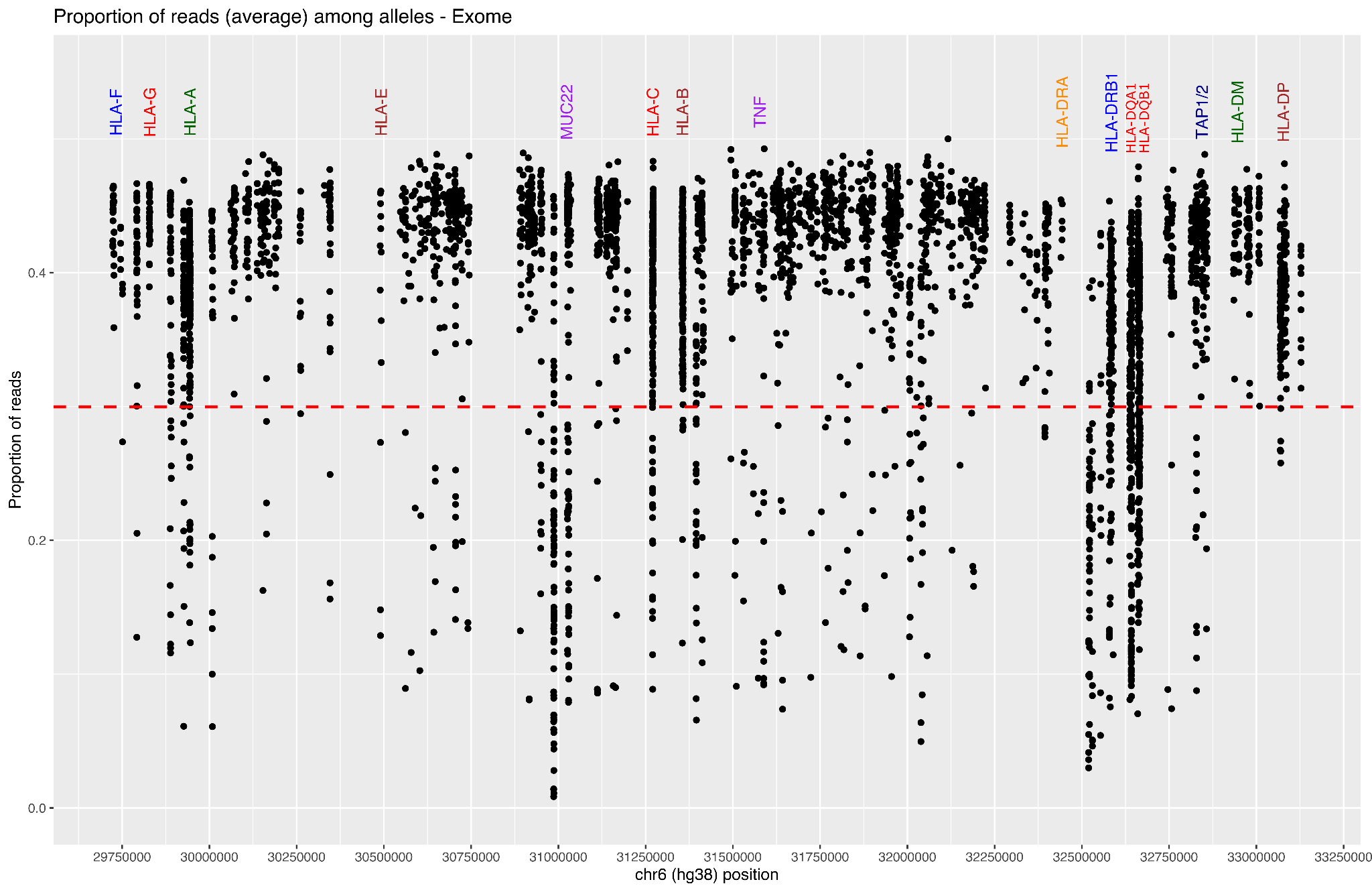


**Supplementary Figure S2:** The proportion of reads (average) among alleles in heterozygous sites for each variant throughout the MHC region detected in exomes from Covid-19 Brazilian patients. We considered only the variants over the red line for comparisons between exomes and whole-genomes.


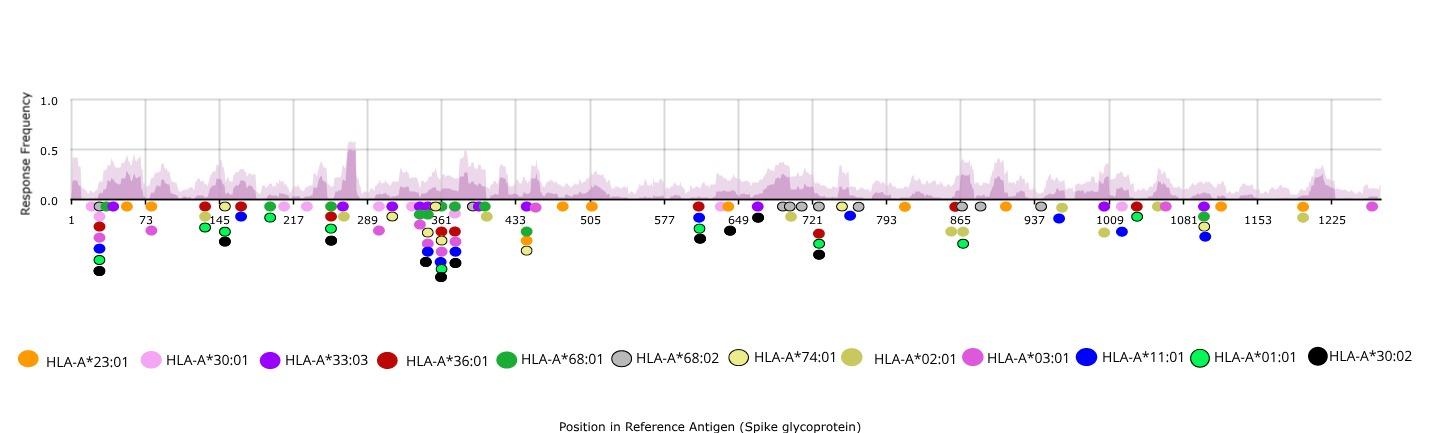


**Figure S3:** Figure 1- Peptides found in the Proteasomal cleavage/TAP transport/MHC class I combined predictor were localized in the map generated with Immunome Browser, containing the immunogenic landscape of SARS-CoV-2 spike protein. Each allele received one color and each peptide is represented as a circle and positioned under the curve of Response frequency depicted with Immunome Browser.


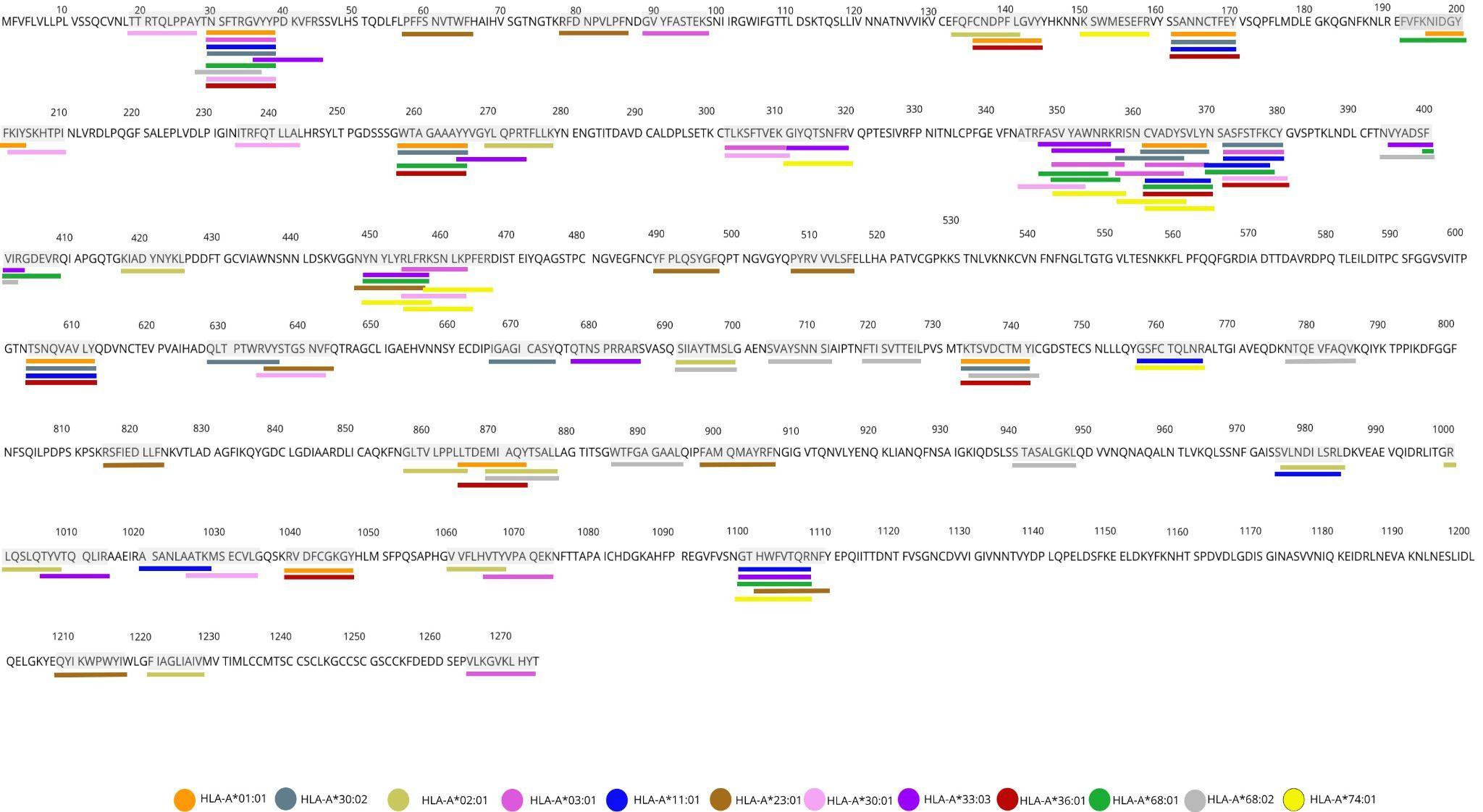


**Supplementary Figure S4:** The sequence of the glycoprotein spike of SARS-CoV-2 was used to identify each peptide found for each allele. The color underlying identifies each allele. It's possible to observe overlapped peptides for different alleles in some parts of the spike.


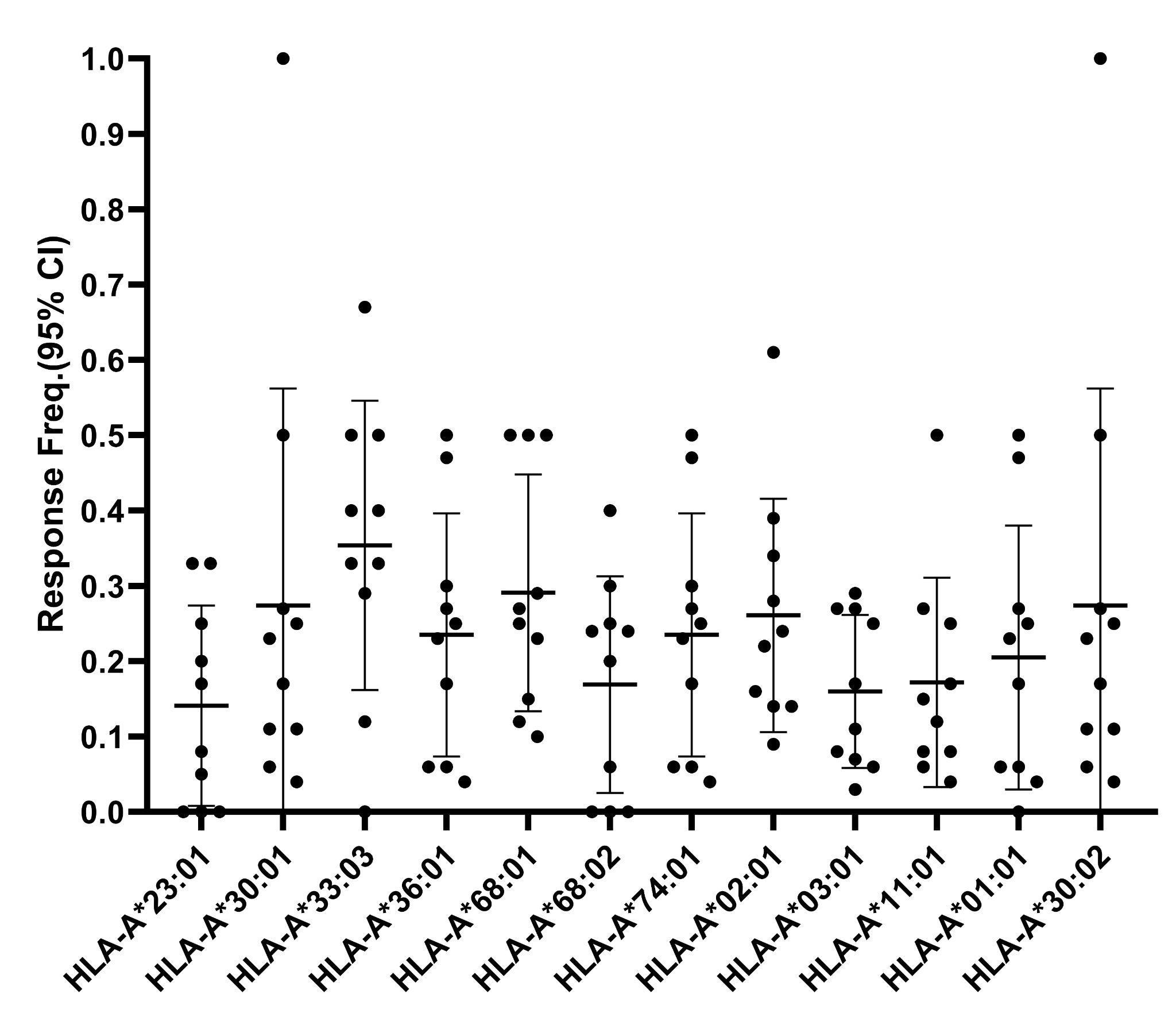


**Supplementary Figure S5:** Response Frequency comparison (95% CI) of 10 selected peptides by MHC-I Processing Predictions between the most frequent alleles found in patients (HLA-A*23:01, HLA-A*30:01, HLA-A*33:03, HLA-A*36:01, HLA-A*68:01, HLA-A*68:02, HLA-A*74:01); alleles related to poor prognosis (HLA-A*01:01 e HLA-A*30:02) or good prognosis (HLA-A*02:01, HLA-A*03:01 e HLA-A*11:01 ). Data are expressed as Response Freq. (95% CI) individual values, mean ± S.D. Statistical analyses by ANOVA Tukey’s multiple comparison test.


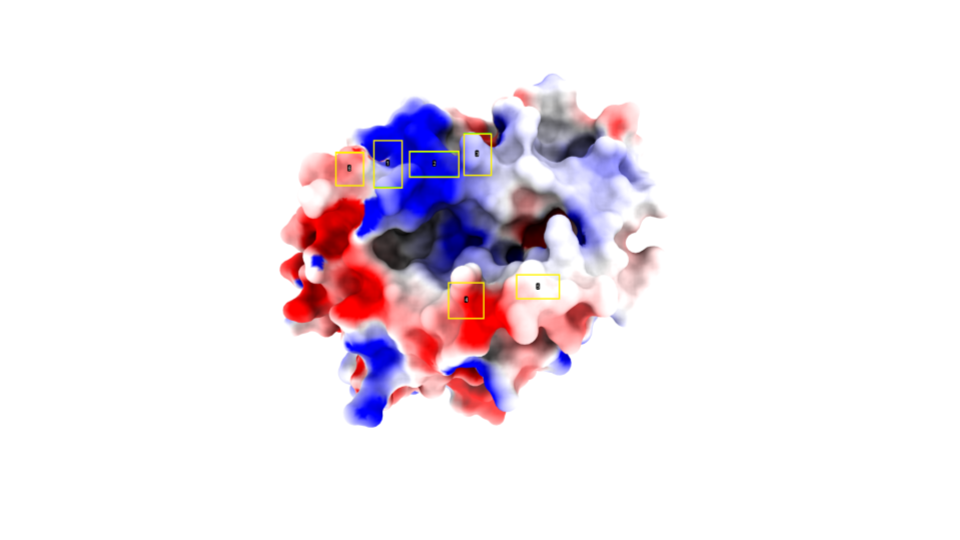


**Supplementary Figure S6:** In an electrostatic surface view of an MHC, the yellow squares are the regions previously selected to extract color information from the image. These regions were chosen based on their rate of contact with the TCR during the immune presentation.

**Supplementary Figure S7:** Haploview plot of linkage disequilibrium (LD) across the MHC region considering SNPs that might be associated with Covid-19 severity. The r^2^ value is represented as shades of gray, with black indicating r^2^ close to 1.0. D' values are represented when different from 1.0.


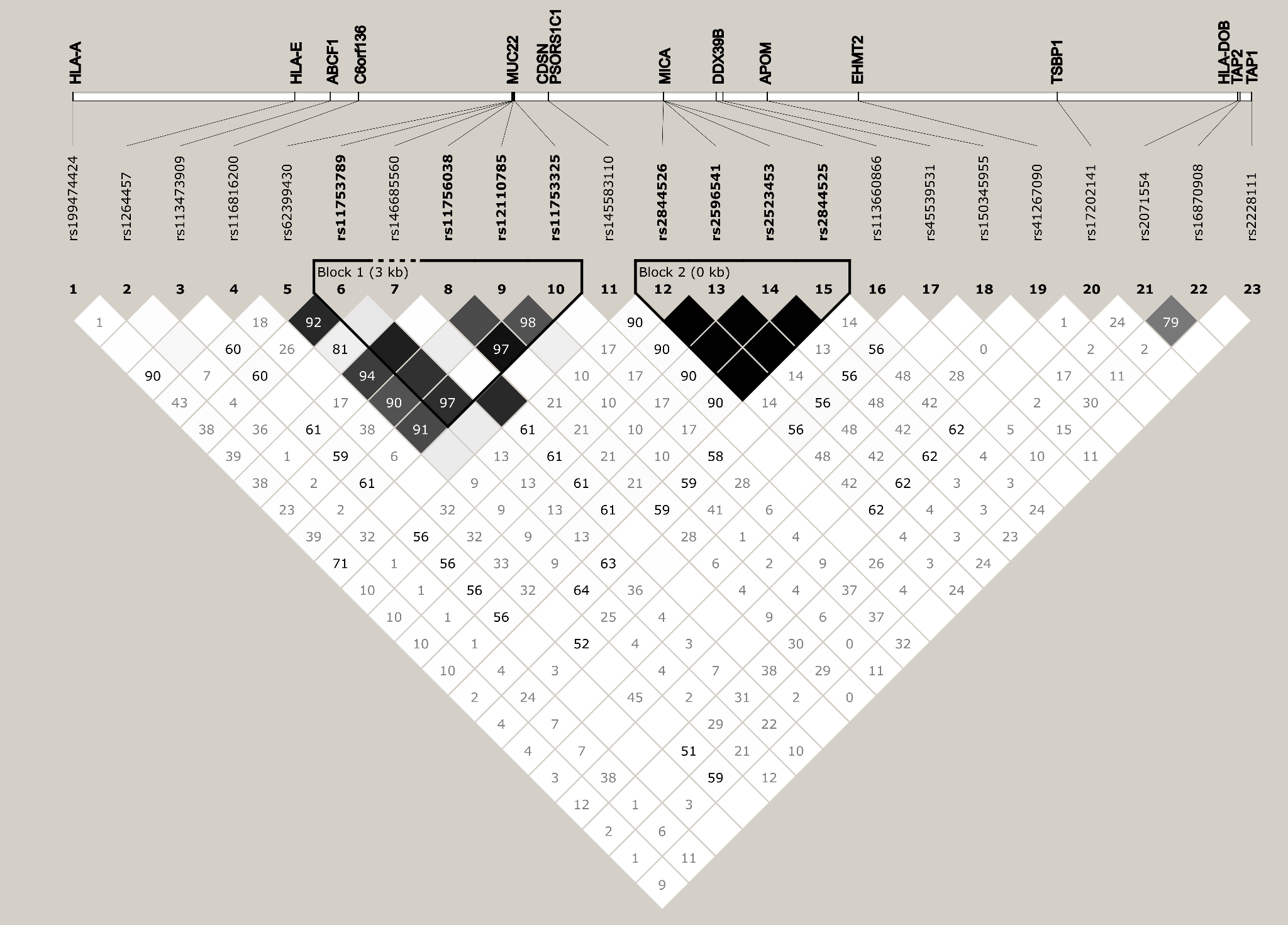


#

## Supplementary Tables

**Supplementary Table S1**: Demographic data and mean genome-wide genetic ancestry for each group

| **Group** | **Size** | **Sex  (% male)** | **Mean age** | **EUR** | **AFR** | **NAM** | **EAS** |
| --- | --- | --- | --- | --- | --- | --- | --- |
| MILD Covid-19 ^a^ | 155 | 41.7 | 66.9 | 86.11 | 8.68 | 4.76 | 0.43 |
| SEVERE Covid-19 | 55 | 56.36 | 51.3 | 63.84 | 21.59 | 14.24 | 0.32 |
| SABE (Brazilian general elderly population) | 1170 | 36.41 | > 65 | 72.63 | 17.85 | 6.69 | 2.79 |
| SUPER ELDERLY recovered from Covid-19 | 87 | 25.28 | 93.8 | 83.17 | 11.06 | 4.58 | 1.17 |

EUR: % European ancestry, AFR: % African ancestry, NAM: % Native American ancestry, EAS: % East-Asian ancestry. ^a^ Covid-19 positive individuals with mild symptoms. This cohort includes the Super elderly with mild symptoms.

**Supplementary Table S2**: IPD Accession and alleles names.

| **IPD Accession** | **Allele Name** |
| --- | --- |
| HLA00001 | A*01:01:01:01 |
| HLA00005 | A*02:01:01:01 |
| HLA00037 | A*03:01:01:01 |
| HLA00043 | A*11:01:01:01 |
| HLA00048 | A*23:01:01:01 |
| HLA00089 | A*30:01:01:01 |
| HLA00106 | A*33:03:01:01 |
| HLA00110 | A*36:01:01:01 |
| HLA00115 | A*68:01:01:01 |
| HLA00117 | A*68:02:01:01 |
| HLA00127 | A*74:01:01:01 |
